# *Mycobacterium tuberculosis* genomic surveillance in Mexico. Characterization of variants in drug resistance and efflux pump genes

**DOI:** 10.3389/fmicb.2025.1666838

**Published:** 2025-10-15

**Authors:** Néstor Alvarado-Peña, Marcela Muñoz Torrico, Luis Narváez-Díaz, Paulina M. Mejía-Ponce, Eduardo Becerril Vargas, Joaquín Zúñiga, Raquel Muñiz-Salazar, Rafael Laniado-Laborín, Cuauhtémoc Licona-Cassani, Xavier Soberón, Eugenia Silva-Herzog

**Affiliations:** ^1^Clínica de Tuberculosis, Instituto Nacional de Enfermedades Respiratorias “Ismael Cosío Villegas”, Mexico City, Mexico; ^2^Laboratorio de Vinculación Científica, Facultad de Medicina-Universidad Nacional Autonoma de Mexico-Instituto Nacional de Medicina Genómica (UNAM-INMEGEN), Mexico City, Mexico; ^3^Laboratorio de Microbiología, Instituto Nacional de Enfermedades Respiratorias (INER), Mexico City, Mexico; ^4^Escuela de Ingeniería y Ciencias, Tecnológico de Monterrey, Monterrey, Mexico; ^5^Laboratorio de Inmunobiología y Genética, Instituto Nacional de Enfermedades Respiratorias “Ismael Cosío Villegas” and Tecnológico de Monterrey, Escuela de Medicina y Ciencias de la Salud, Mexico City, Mexico; ^6^Escuela de Ciencias de la Salud, Universidad Autónoma de Baja California, Ensenada, Mexico; ^7^Facultad de Medicina, Universidad Autónoma de Baja California, Tijuana, Mexico; ^8^Tecnológico de Monterrey, The Institute for Obesity Research, Monterrey, Mexico; ^9^Departamento de Ingeniería Celular y Biocatálisis, Instituto de Biotecnología, Universidad Nacional Autónoma de México, Cuernavaca, Mexico

**Keywords:** tuberculosis, *Mycobacterium tuberculosis* complex, drug-resistant tuberculosis, resistance-associated variants, efflux pumps, whole genome sequencing

## Abstract

Tuberculosis (TB) remains a persistent global public health challenge, with the rise of drug-resistant tuberculosis (DR-TB) complicating all the disease control efforts. The World Health Organization (WHO) has advocated for molecular diagnostic techniques, including whole-genome sequencing (WGS), to enhance TB diagnosis and treatment strategies. In this study, we performed WGS analysis on 49 pulmonary tuberculosis isolates from Mexican patients to identify mutations conferring resistance to 11 key antimicrobial agents: four first-line drugs (isoniazid, rifampicin, ethambutol, and pyrazinamide) and 7 second line drugs (fluoroquinolones, ethionamide/prothionamide, amikacin, kanamycin, capreomycin, streptomycin, and bedaquiline). We identified 89 novel variants: 48 in genes previously associated with drug resistance and 41 in genes not previously linked to resistance mechanisms, including potential novel mutations associated with delamanid resistance. Additionally, we detected 31 mutations across three efflux pump superfamilies (ABC, RND, and MFS); all of these variants warrant further investigation regarding their contribution to antibiotic resistance. This analysis represents approximately 10% of Mexico’s national variant registry, providing substantial insight into the molecular epidemiology of drug-resistant tuberculosis within the country. The identification of new resistance-associated variants (RAV) from clinical isolates underrepresented in global databases, contributes to develop improved diagnostic tools, optimize treatment regimens, and probably to elucidate antibiotic resistance mechanisms. Specifically, the identification of RAVs for new drugs like bedaquiline, pretomanid, delamanid, and linezolid, which are central to the most recent schemes of treatment (BPaLM, BPaL, BDLLfxC, BLMZ), is key to the improvement of patient outcomes and preventing the emergence of resistance to these critical therapeutic options.

## 1 Introduction

Tuberculosis (TB) is an infectious disease caused by the *Mycobacterium tuberculosis* Complex (*MtbC*) that remains one of the 13 leading causes of death worldwide. After the COVID-19 pandemic, TB returned as the principal cause of death for a single infection agent, with 10.8 million new cases and 1.25 million deaths worldwide (Global Tuberculosis Report, 2024).

TB can be caused by drug-susceptible (DS) or drug-resistant (DR) strains. DR-TB treatment has the worst prognosis, which in general results in longer treatment durations, increased adverse effects for patients, and higher costs. The rise of rifampicin-resistant TB (RR-TB) is a global threat that has led the World Health Organization (WHO) to classify it as a priority pathogen (World Health Organization, 2024b). DR-TB can lead to Multidrug Resistance (MDR-TB), defined by WHO as Resistance to Rifampicin (RR) and isoniazid (H), or Extensive Drug Resistance (XDR-TB), defined as resistant to rifampicin (may also be resistant to isoniazid) plus a fluoroquinolone (levofloxacin or moxifloxacin), and either bedaquiline or linezolid. Worldwide incidence of MDR/RR-TB has decreased from an estimated 580,000 cases (UI 460,000 -580,000) in 2015 to 400,000 (UI 360,000 – 440,000) in 2023, mainly because of better diagnosis and prompt detection. However, the incidence of Pre-XDR and XDR TB has increased 3.8-fold in this same time period (Global Tuberculosis Report, 2016; Global Tuberculosis Report, 2024).

Rapid and accurate detection of resistant profiles is crucial for successful treatment, reducing transmission, as well as the risk of an increased proportion of resistant strains (Roberts et al., 2024). Overall, bacteria employ diverse mechanisms to resist antibiotic effects, including the enzymatic degradation of antibiotic molecules, acquisition of mutations within or around drug targets, reduced membrane permeability, and overexpression of efflux pumps. These resistance mechanisms leave distinctive genetic signatures that can be localized in specific genomic regions or distributed throughout the entire genome. Next-generation sequencing technologies, including amplicon sequencing and whole-genome sequencing (WGS), offer the potential to identify resistance markers and generate comprehensive antimicrobial resistance profiles (World Health Organization, 2024a; Pérez, 2023).

The identification and validation of DR markers are the result of extensive genomic studies coupled with microbiological tests that have been performed mainly in regions with high disease prevalence. These studies focus mainly on drug targets and drug metabolism, with less emphasis on efflux pumps (The CRyPTIC Consortium, 2022; World Health Organization, 2023). The importance of efflux pumps in antibiotic resistance is becoming more apparent in recent years (Li et al., 2024; Gaurav et al., 2023). They help maintain cellular homeostasis by expelling toxic molecules, which keeps antibiotic concentrations below therapeutic levels. Therefore, efflux pumps should be included in any genetic analysis of antibiotic resistance.

While these global studies encompass a few strains from Latin America, very few come from Mexico. Although the WHO considers Mexico a low-burden country for TB, it ranks among the top three countries with the most DR-TB in the Americas, together with Brazil and Peru (Tuberculosis en las Américas, 2021; Global Tuberculosis Report, 2024). DR-TB cases in Mexico have increased 281% in recent years (from 283 in 2015 to 796 reported in 2023; National Center for Preventive Programs and Disease Control, 2024), stressing the importance of antibiotic surveillance in Mexico.

The majority of genomic epidemiological studies from Mexican TB patients are centered on mutations for first-line drugs, including rifampicin, isoniazid, ethambutol, pyrazinamide, aminoglycosides, and fluoroquinolone, but do not explore mutations for drugs recently introduced in treatment regimens by WHO, such as bedaquiline, linezolid, pretomanid and delamanid, which are now a central part of TB treatment (Zenteno-Cuevas et al., 2009; Lopez-Alvarez et al., 2010; Flores-Treviño et al., 2015; Zenteno-Cuevas et al., 2014; Juarez-Eusebio et al., 2017; Madrazo-Moya et al., 2019; Zenteno-Cuevas et al., 2020; Mónica et al., 2021; Barbosa-Amezcua et al., 2022; Mejía-Ponce et al., 2023). In this study, we aim to characterize both known and novel mutations in target genes and efflux pump genes associated with resistance in Mexican patients with pulmonary-resistant tuberculosis using WGS. For strains with available phenotypic data, we analyze genotype–phenotype correlations. A solid knowledge of the genetic characteristics of resistance will help develop precise and maybe individual treatments to better help patients with DR-TB.

## 2 Materials and methods

### 2.1 Clinical sample collection

A collection of 61 clinical isolates with confirmed drug resistance by phenotypic drug sensitivity test (pDST) was selected from the Mycobacterial laboratory at the Instituto Nacional de Enfermedades Respiratorias (INER), a national reference center for TB in Mexico, and from the *Mycobacterium tuberculosis* collection at the Universidad Autónoma de Baja California (UABC). The study prioritized samples with the highest resistance profiles (22 MDR and 11 Pre-XDR strains) from these collections, representing nine Mexican states over 9 years (2014–2023). Of the 61 original isolates, 12 strains could not be included because either they were impossible to subculture or insufficient DNA was obtained for analysis, resulting in 49 total strains in the study.

The samples were decontaminated using Petroff’s modified method before taking an aliquot and grown in BACTEC MGIT liquid culture (Becton Dickinson, Sparks, United States). Microbiological drug susceptibility tests were performed at the time of first isolation following the proportion method, to find the lowest drug concentration that inhibits 99% of bacterial growth. Standard drug concentrations tested include isoniazid (0.1, 0.4, 1.0, 4.0 μg/mL), rifampicin (0.5, 1.0 μg/mL), ethambutol (5.0, 7.5, 8.0 μg/mL), pyrazinamide (100.0 μg/mL), amikacin (1.0, 2.0 μg/mL), kanamycin (2.5, 5.0 μg /mL), capreomycin (0.25, 5.0 pg./mL), clofazimine (1.0 μg/mL), ethionamide (5.0 μg/mL), streptomycin (1.0, 4.0 μg/mL), levofloxacin (1.0 μg/mL), moxifloxacin (0.25, 1.0, 2.0 μg/mL), ofloxacin (2.0 μg/mL), linezolid (1.0 μg/mL), and cycloserine (40.0 μg/mL). All strains were heat-inactivated in a dry bath at 95 °C for 30 min and sent in triple packaging to the “Instituto Nacional de Medicina Genómica” (INMEGEN) for DNA extraction and bioinformatic analysis. Transportation and processing was conducted in adherence with the biosafety recommendations established by the INER and INMEGEN.

### 2.2 DNA extraction and whole genome sequencing

DNA extraction was performed using the QIAmp UCP Pathogen Kit (Qiagen) according to the manufacturer’s recommendations. The quality and concentration of DNA were evaluated using NanoDrop (ThermoFisher) in the laboratory, as well as Qubit Fluorometer, and agarose Gel Electrophoresis Quantitation at Novogene Corporation (Novogene Co., Sacramento, CA). DNA library preparation and sequencing for microbial WGS were performed on the NovaSeq PE150 platform (Illumina) at Novogene Corporation (Novogene Co., Sacramento, CA).

### 2.3 Bioinformatics analysis

The bioinformatic analysis of the sequences was conducted using the methodology described by Cuevas-Córdoba et al. (2021). Briefly, removal adapters and low-quality reads (<30 Phred-scaled) were followed by the elimination of human, viral, and other bacterial sequences identified with SURPI (“Sequence-based ultrarapid pathogen identification”) (Naccache et al., 2014). Reads were then mapped to the Mtb reference strain H37Rv genome (RefSeq Accession: NC_000962.3). An overall genome coverage of > 278 fold (65–399 fold) was achieved. Variant calling relied on the GATK software with annotations provided by SnpEff software. Variants found in our analysis were classified as “Associated with Resistance,” “Not Associated with Resistance,” and “Uncertain” following a compiled database that includes WHO’s “Catalog of mutations in *Mycobacterium tuberculosis* complex and their association with drug resistance” 2nd edition (2023) and The CRyPTIC Consortium (2022). All variants excluded from these datasets were classified as “No Information.” A table with compensatory mutations was made based on reported studies (Comas et al., 2012; Conkle-Gutierrez et al., 2023; Napier et al., 2023; Billows et al., 2024). Furthermore, drug-efflux pumps previously associated with phenotypic resistance to antibiotics (Pal et al., 2014; Narang et al., 2017; Ghajavand et al., 2019; Raheem et al., 2020; Rodrigues et al., 2020; Laws et al., 2022; Hasan et al., 2024; Rao and Bhosale, 2024) were used to create an additional database. The lineage of MTB strains was determined using the software Mykrobe.^1^

#### 2.3.1 Phylogenetic reconstruction

Quality-filtered reads were analyzed with MTBseq v.1.1.0 (Kohl et al., 2018) to generate a whole genome-based SNP alignment for phylogenetic inference under default parameters. The number of invariant sites in the alignment was calculated using the MTBseq_to_phylo.py script (available at GitHub - conmeehan/pathophy: Scripts for aiding in pathogen phylogenetics analysis). Phylogenetic trees were reconstructed with RAxML-NG v.1.2.2, applying the --model GTR + G + ASC_STAM {757,600/1447853/1442541/757415} and --site-repeat options. Bootstrap convergence was achieved after 150 replicates, supporting the tree topology. Tree visualization was performed in iTOL v7. Sub-lineage assignment and drug-resistance classification were obtained with TBprofiler v.6.6.5 (Phelan et al., 2019), using the --itol flag to export the corresponding tree annotation files. *Mycobacterium microti* served as the outgroup for tree rooting.

All sequences and metadata are available at the NCBI database under the BioProject_PRJNA1260069.

### 2.4 Statistical analysis

All statistical analyses were performed using SPSS v 25 (IBM SPSS Statistics, version 25). Clinical and demographic variables with normal distribution were expressed as median ± standard deviation (SD). Categorical variables were presented as frequencies (percentages) and compared using Fisher’s exact test; a two-tailed *p* value of <0.05 was considered statistically significant. Genotypic resistance for each drug was compared with the corresponding pDST. Sensitivity, specificity, positive predictive value (PPV), and negative predictive value (NPV) were calculated using a clinical calculator (Merck & Co, Inc., Rahway, NJ, United States, 2025). The strength of agreement between phenotype and genotype was determined with Cohen’s kappa coefficient. The Cohen’s Kappa values indicate agreement and are interpreted as follows: values ≤ 0 indicating no agreement, 0.01–0.20 as none to slight, 0.21–0.40 as fair, 0.41–0.60 as moderate, 0.61–0.80 as substantial, and 0.81–1.00 as almost perfect agreement (McHugh, 2012). The graphs were generated using R (R Version 2024.09.01 + 394, packages basic, ggplot2, reshape2, and tidyr).

### 2.5 Ethical concerns

The project was approved by the Ethical Committees from both, INER (Comité de ética en investigación INER) and UABC (Comité de ética en investigación UABC). Written informed consent was obtained from all participants before the collection of samples and the recording of clinical data; an exemption was obtained for older samples. All the information was treated confidentially.

## 3 Results

### 3.1 Population characteristics

The samples for this study encompass mostly central and northern Mexico and include Ciudad de Mexico (24%), Baja California (18.5%), Estado de Mexico (16.6%), Guerrero (43.2%), Veracruz (9.2%), Puebla (5.5%), Hidalgo, Zacatecas, and Tamaulipas (each 1.8%). We specifically chose the highest resistant profile in the collections, focusing on MDR and Pre-XDR strains. Overall, the mean age of the patients was 42.5 years (range, 17–81), and 61.3% were men. The most common comorbidity was Type-2-Diabetes (36.7%), and 8% were living with HIV. Half of the patients had previous treatment at the time of sample collection, and 26% had no treatment recorded (see Table 1 for the full description of epidemiological data of the population sampled).

**Table 1:** Demographic and clinical characteristics of study participants.

Sex	Number (% of total)
Female	17 (34.7%)
Male	30 (61.3%)
Unknown	2 (4.0%)
Age Median (42.48), 15.73 sd	
<18	1 (2%)
19–29	10 (20.4%)
30–44	15 (30.6%)
45–64	17 (34.7)
>65	4 (8.2%)
Unknown	2 (4.0%)
BMI Median (21.45), 3.62 sd	
< 18.5	10 (20.4%)
18.5–24.99	24 (49%)
≥25.00- < 30.00	6 (12.2%)
Unknown	9 (18.4%)
Drug resistance	
Susceptible	3 (6.12%)
Polydrug resistant	3 (6.12%)
Hr-TB	3 (6.12%)
RR-TB	3 (6.12%)
MDR-TB	22 (44.9%)
pre-XDR-TB	11 (22.4%)
Unknown	4 (8.16%)
Diabetes Type 2	
Yes	18 (36.7%)
No	21 (42.8%)
Unknown	10 (20.4%)
Living with HIV	
Yes	4 (8.16%)
No	36 (73.4%)
Unknown	9 (18.36%)
Previous TB treatment	
Yes	27 (55.10%)
No	13 (26.5%)
Unknown	9 (18.36%)

Number of strains (% of total).

The pDST included first and second line antibiotics, and revealed distinct drug-resistant profiles in our samples, including 44.9% of MDR, and 22.4% Pre-XDR samples, according to WHO classification criteria.

### 3.2 Genotypic analysis of clinical samples

An average of 8.8 Million high-quality reads per sample were obtained, providing an average coverage depth of 278.3 times (65–399) of the reference genome. WGS revealed that all our samples belong to the Euro-American lineage (lineage 4), except for two, which were identified as *Mycobacterium bovis*. The most prevalent sublineages are 4.1.2.1 (“Harlem”), 4.4.1.1, 4.1.1.3 (“X3”), and 4.10 (20.4, 18.39, 18.36, and 14.28% respectively).

Genotypic analysis of this population revealed that 89.8% of the samples were predicted to be resistant to at least one of the first-line drugs (rifampin, isoniazid, ethambutol, and pyrazinamide). Twenty-nine samples (59%) were classified as MDR/RR, and 12 samples, or 24.5%, were predicted to be resistant to second-line drugs (levofloxacin, moxifloxacin, bedaquiline).

**Figure 1:**
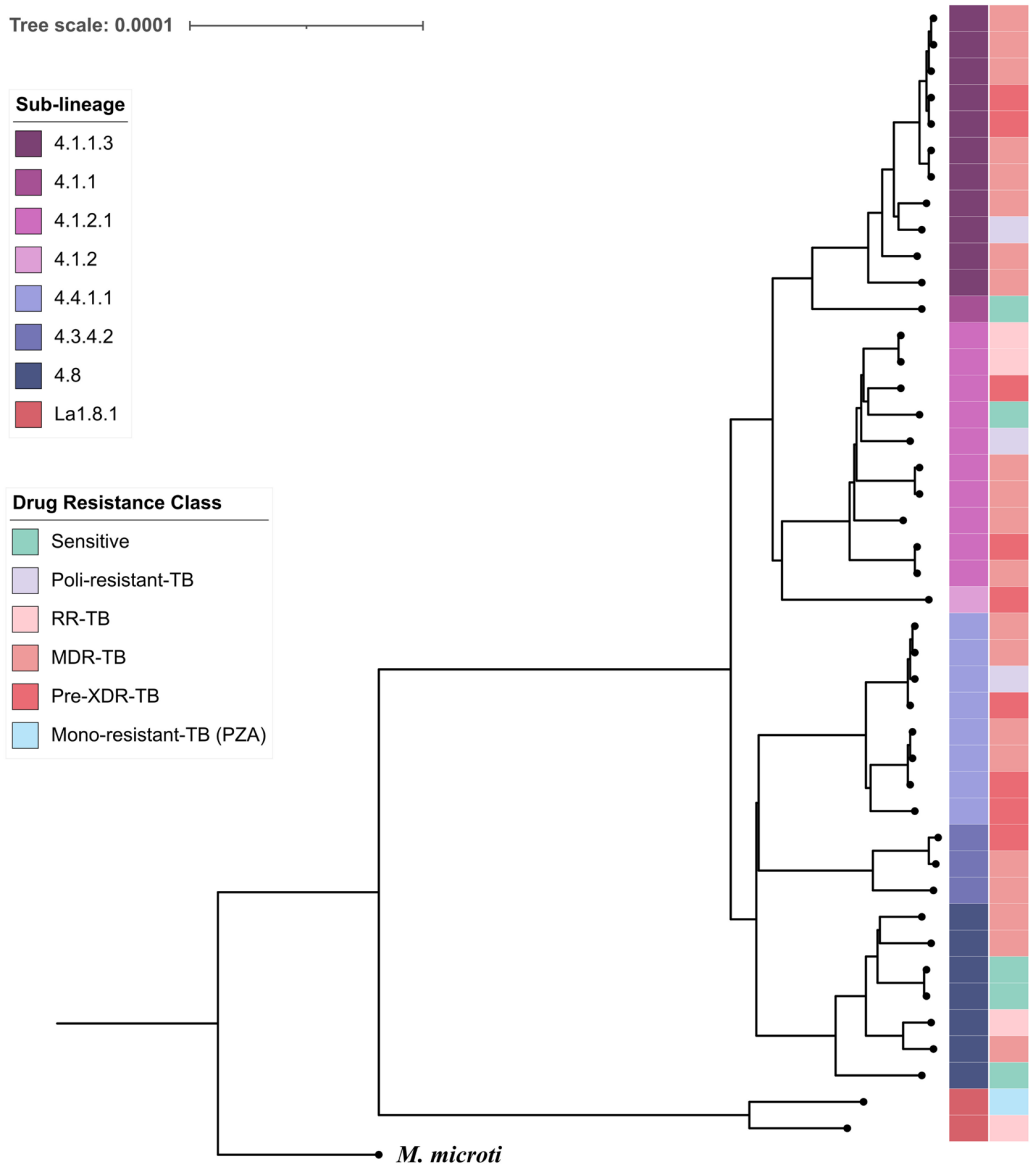
SNP-based phylogeny of MTBC isolates. The tree was inferred from 6,123 variable sites. Convergence was reached after 150 bootstrap replicates, meeting the standard cutoff of 0.03. Branches are annotated by sub-lineage and drug-resistance profile as determined by TB-profiler. *Mycobacterium microti* was included as the outgroup to root the tree.

Whole-genome SNP alignment comprising 6,123 variable sites (Figure 1) was used for phylogenetic reconstruction. A maximum-likelihood tree rooted with *M. microti* revealed well-defined clustering by sub-lineage; branch annotations indicate lineage assignment and predicted drug-resistance profiles, supporting an association between sublineage X3 and increased drug resistance.

**Figure 2:**
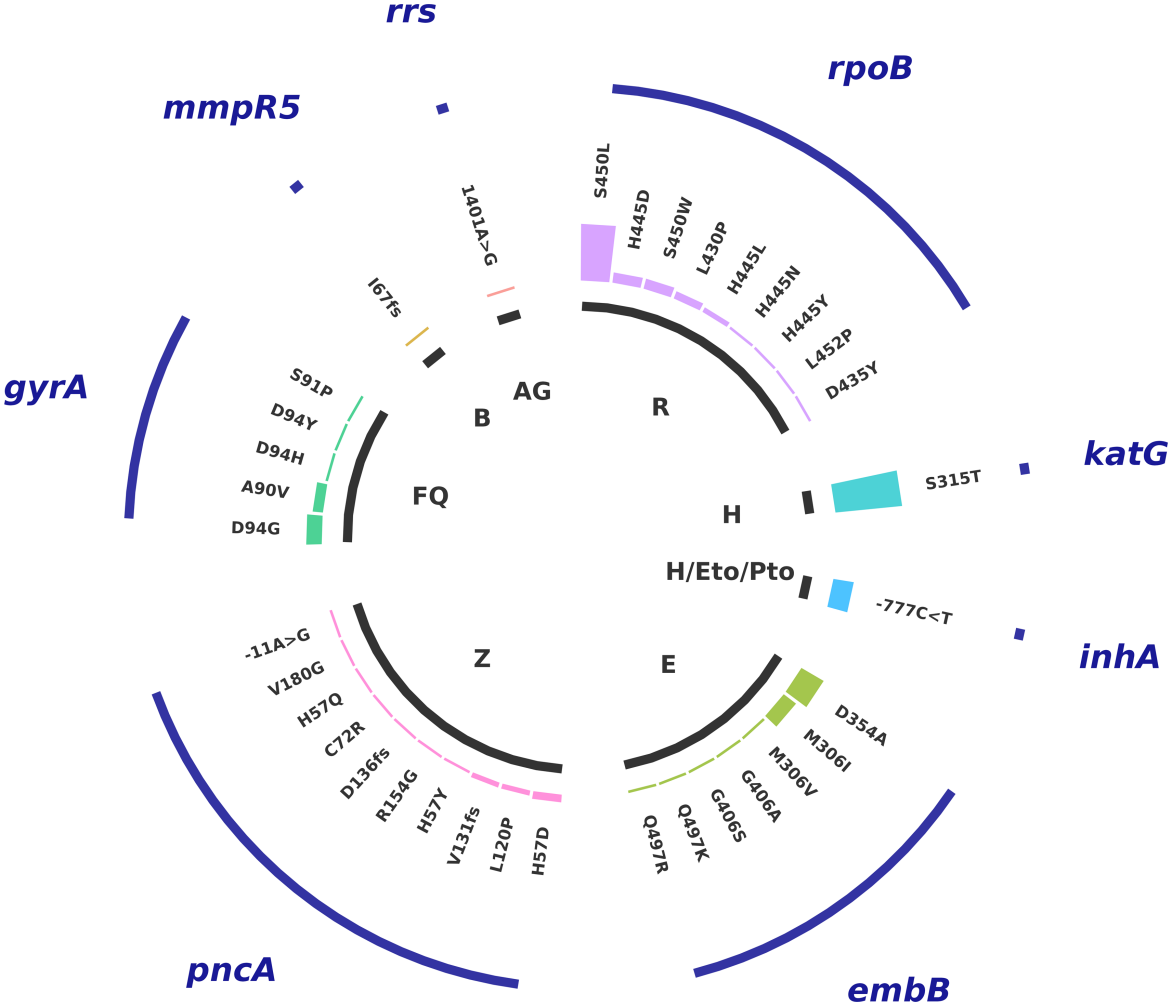
Distribution of mutations associated with resistance. Variants in genes associated with resistance to first-line and second-line antituberculosis drugs: R: rifampicin, H: isoniazid, Eto/Pto: ethionamide/proteonamide, E: ethambutol, Z: pyrazinamide, FQ: fluoroquinolones (moxifloxacin, levofloxacin), B: Bedaquiline, AG: aminoglycosides.

Overall, the most frequent mutations are *katG*_S315T (53% of all samples), *rpoB*_S450L (45%), and *embB*_D354A (20%), variants that confer resistance to isoniazid, rifampicin, and ethambutol, respectively (Figure 2). This corresponds well with the prevalence of antibiotic resistance profile of MDR in our sample population. Among the next most frequent mutations, those affecting resistance to second-line drugs include *inhA*_-777C > T (16%), which confers cross-resistance to isoniazid and prothionamide/ethionamide (Pto/Eto), and *gyrA*_D94D (12%), which confers resistance to levofloxacin and moxifloxacin, and reflects the Pre-XDR antibiotic resistance profile. We also found one strain with an *rrs*_517C > T mutation, which confers resistance to streptomycin, a virtually obsolete treatment option for TB, and another with a *rrs_*1401A > G mutation that confers resistance to kanamycin, capreomycin, and amikacin. Additionally, we identified one strain containing the variant *mmpR5*_I67fs, which confers resistance to bedaquiline with no cross-resistance to clofazimine (World Health Organization, 2023; see Figure 2).

WGS allowed us to identify 89 variants not reported on consensus databases (World Health Organization, 2023; The CRyPTIC Consortium, 2022; see Supplementary Table 1).

Furthermore, we identified 15 compensatory mutations (Table 2), which are second site mutations that reduce the fitness cost associated with antibiotic resistance phenotypes, therefore allowing resistance genes to be stably maintained in bacterial populations. We found that eight strains carrying the *rpoB*_S450L mutation had acquired previously reported compensatory mutations in the RNA polymerase subunit RpoC (V483G/A and I491V) (Comas et al., 2012; Conkle-Gutierrez et al., 2023; Table 2). We also detected four isoniazid-resistant strains with compensatory mutations in *ahpC*_-81C > T, *ahpC*_-52C > A, *ahpC*_-51G > A, and *ahpC*_-47_-46ins (Napier et al., 2023; Billows et al., 2024).

**Table 2:** Mutations identified as compensatory.

Drug	Gen	Variants	Number of strains with this variant
Rifampicin	*rpoC*	V483G	6
		I491V	2
		V483A	1
Isoniazid	*ahpC*	-81C > T	2
		-52C > A	2
		-51G > A	1
		-47_-46insT	1

Compensatory mutations to rifampicin and isoniazid, identified in sample population.

Intriguingly, the majority of our samples contained variants in *gyrA*, with 98% exhibiting the E21Q mutation and 81% also carrying S95T and G668D mutations, classified as “Not associated with Resistance.” Additionally, 46% contained the *embC*_V981L variant and 20% *mshA*_N111S, also classified as “Not Associated with Resistance.” We identified 10 strains with mutations in genes recently associated with delamanid resistance: *dprE2, fbiA, fbiB,* and *ddn*. However, these specific variants (*dprE2*_D45N, *fbiA*_I208V, *fbiB*_G19E, and *ddn*_18G > A) lack a confirmed link to resistance and require validation (Supplementary Table 2; World Health Organization, 2023).

### 3.3 Genotypic and phenotypic drug resistance analysis

Next, we compared the genotypic resistance profile for each drug to pDST results when available. Table 3 shows high sensitivity (>80%) to first-line drugs (rifampicin, isoniazid, and ethambutol), except for pyrazinamide (<50%). Specificity, PPV, NPV, and accuracy were high (>70%) for all drugs analyzed except rifampicin. Cohen’s kappa coefficient showed good concordance (0.8–1.0) for ethionamide; good agreement (0.6–0.79) between phenotypic and genotypic results for isoniazid, ethambutol, moxifloxacin, and levofloxacin; moderate agreement (0.4–0.59) for pyrazinamide, amikacin, and capreomycin.

**Table 3:** Phenotypic and genotypic drug resistance concordance analysis.

Drugs	Phenotypic resistance	Genotypic resistance	Sensitivity	Specificity	PPV^a^	NPV^b^	Cohen’s Kappa (95% CI)
Rifampicin	36/49	38/49	88.90%	66.70%	4.48%	99.68%	**0.533**, P 0.002 (0.001–0.002)
Isoniazid	38/49	34/49	86.80%	100%	100%	99.75%	**0.672**, P 0.000 (0.000–0.000)
Pyrazinamide	20/49	14/49	45.00%	95.70%	16.69%	98.91%	**0.420**, P 0.003 (0.002–0.005)
Ethambutol	17/49	22/49	88.20%	85.70%	10.56%	99.74%	**0.723**, P 0.000 (0.000–0.000)
Streptomycin	17/49	1/49	5.90%	100%	100%	98.23%	**0.067**, **P 0.430** (0.420–0.440)
Moxifloxacin	10/49	11/49	80%	90.90%	14.40%	99.58%	**0.685**, P 0.000 (0.000–0.000)
Levofloxacin	4/49	11/49	100%	90.90%	17.38%	100%	**0.755**, P 0.001 (0.000–0.002)
Kanamycin	4/49	1/49	25%	100%	100%	98.58%	**0.341**, **P 0.226** (0.218–0.234)
Amikacin	3/49	1/49	33.30%	100%	100%	98.74%	**0.480**, P 0.075 (0.070–0.080)
Capreomycin	3/49	1/49	33.30%	100%	100%	98.74%	**0.457**, **P 0.161** (0.154–0.168)
Etionamid	2/49	8/49	100%	100%	100%	100%	**1.0**, P 0.004 (0.003–0.005)

^a^PPV Positive Predictive Value. ^b^NPV Negative Predictive Value.

### 3.4 Variant analysis in efflux pumps

Efflux pump mechanisms, both multidrug and drug-specific efflux pump mechanisms, are important determinants of antimicrobial resistance. Therefore, we specifically searched for efflux pumps previously identified as associated with antibiotic resistance (Domenech et al., 2005; Pal et al., 2014; Anthony Malinga and Stoltz, 2016; Narang et al., 2017; Ghajavand et al., 2019; Melly and Purdy, 2019; Raheem et al., 2020; Rodrigues et al., 2020; Laws et al., 2022; Remm et al., 2022; The CRyPTIC Consortium, 2022; Hasan et al., 2024). In this analysis, we found that 73.4% of our samples encoded variants in members of the ABC, RND, and MFS superfamilies but none in the SMR or MATE efflux pump superfamilies. Overall, the most frequent variants were found in the ABC and RND superfamilies, present in 47 and 45% of all our samples, respectively. Within the ABC superfamily, the most frequent variant was on Rv1458c (present in 39% of our samples), which has been associated with resistance to rifampicin, isoniazid, ethambutol, and streptomycin (Hao et al., 2011; Laws et al., 2022). In addition, we identified 15 strains (30% of our samples) with mutations on *mmpL8* member of the RND superfamily associated with glycolipid transport and isoniazid resistance, nine on *mmpL3* associated resistance to bedaquiline, clofazimine, linezolid, and delamanid (Domenech et al., 2005; Melly and Purdy, 2019; Laws et al., 2022; The CRyPTIC Consortium, 2022; see Table 4).

**Table 4:** Mutations in efflux pumps.

Family	Efflux pumps	Variants	Number of strains with this variant	Substrates	References
RND superfamily	MmpL1	T363M	3	Fatty acid transport	Melly and Purdy (2019)
	MmpS1	I122N	1	Unknown	Not Apply
	MmpL3	D466E	3	Bedaquilne, Clofazimine, Linezolid, Delamanid. Glycolipid transport	The CRyPTIC Consortium (2022) and Adams et al. (2021)
		F384I	3		
		A362S	3		
		G747R	1		
		T284A	1		
		P791L	1		
	MmpL8	A1042E	3	Isoniazid Sulfated Glycolipid	Hasan et al. (2024) and Narang et al. (2017)
		V258A	2		
		V961M	2		
		V526A	2		
		G235R	2		
		V728L	1		
		L56fs	1		
		L788L	1		
		G55V	1		
		G692E	1		
		64C > T	9		
ABC Superfamily	Rv1458c	P5fs	16	Rifampicin, Isoniazid, Ethambutol, Streptomycin	Hasan et al. (2024), Laws et al. (2022), and Anthony Malinga and Stoltz (2016)
		V1_A4del	2		
	Rv1819c/BacA	I603V	3	Rifampicin, Isoniazid, Aminoglycosides, Antimicrobial peptides, Beta-lactams, Chloramphenicol, Macrolides, Novobiocin, Tetracycline, Vancomycin	Laws et al. (2022) and Li et al. (2024)
		L551fs	1		
		D379G	1		
	Rv0342/IniA	N88S	2	Rifampicin, Ethambutol	Raheem et al. (2020)
		P3A	2		
		H481Q	1		
		A374V	1		
MFS Superfamilia	Rv2846c/EfpA	T15R	3	Rifampicin, Isoniazid, Fluoroquinolones, Acriflavine, Erythromycin, Ethidium bromide	Rodrigues et al. (2020) and Laws et al. (2022)
	Rv2459/JefA	M55I	2	Rifampicin, Isoniazid, Ethambutol, Ethidium bromide	Rodrigues et al. (2020) and Laws et al. (2022)
		L44fs	1		
		A419T	1		
	Rv1410c/P55	M182L	1	Rifampicin, Isoniazid, Aminoglycosides, Clofazimine, Tetracyclines.	Rodrigues et al. (2020) and Laws et al. (2022)

Variants on efflux pumps genes, number of strains with variants; substrates reported for each efflux pump.

Most of the strains (67.5%) with mutations in efflux pumps had only one mutation, 21.6% had variants in two different efflux pumps, and two strains had five different efflux pumps mutated; these two strains were identified as *M. bovis* and had the lowest congruency between phenotypic tests and genotypic characterization.

## 4 Discussion

The increasing incidence of DR-TB has reduced the number of effective antibiotics, thus complicating the efforts to combat this persistent global health problem. Drug resistance phenotypes are acquired through the accumulation of mutations (single-nucleotide polymorphisms (SNPs), insertions, or deletions) in genes that code for drug targets or enzymes that activate drugs. This study presents WGS characterization of 49 resistant clinical isolates with at least first-line pDST collected from central and northern Mexico from 2014 to 2023. Among these isolates, 44.8% were classified as MDR-TB and 22.4% as Pre-XDR.

The rate of mutation and the effect of these variants on drug resistance are sometimes dependent on the genetic background of the *Mtb* strain (Ford et al., 2013; Nimmo et al., 2022). Therefore, it is crucial to characterize the resistance patterns in various regions, with their corresponding prevalent strains, to evaluate and design effective treatment strategies. Latin America has few studies examining resistance variants, although more interest has been given in recent years to our population (Zenteno-Cuevas et al., 2014; Madrazo-Moya et al., 2019; Jiménez-Ruano et al., 2021; Santos-Lazaro et al., 2021; Barbosa-Amezcua et al., 2022; D’Souza et al., 2023; Mejía-Ponce et al., 2023; Morey-León et al., 2023; Conceição et al., 2024; Puyén et al., 2024). It is important to emphasize that DR-TB reporting in Mexico has increased significantly over the last 4 years, after obvious underreporting due to health services being redirected toward COVID-19 pandemic response. DR-TB cases increased from 283 to 796 from 2015 to 2023, in contrast with global DR-TB trends that have remained relatively stable or decreased over the same period (National Center for Preventive Programs and Disease Control, 2023; Global Tuberculosis Report, 2024). Thus, a robust epidemiological surveillance system, based on local data, is crucial for accurate diagnosis and enhanced treatment, which could enable the identification of areas for improvement of health policies to prevent transmission.

The available evidence consistently shows lineage 4 as the dominant clade (>90% of the isolates) in Mexico. National surveys based on WGS and genotyping confirm the expansion of Haarlem (4.1.2.1), X-type (notably sub-lineage 4.1.1.3, “X3”), and 4.8 across several Mexican states. Published work (e.g., Jiménez-Ruano et al., 2021; Mejía-Ponce et al., 2023) reports that X3 is primarily restricted to Mexico and is disproportionately enriched for multidrug-resistant strains. Other L4 sub-lineages such as 4.1.2.1 and 4.8 have also been linked to elevated drug-resistance frequencies in regional cohorts (Molina-Torres et al., 2022; Alvarez-Maya et al., 2025). These findings indicate that, while L4 predominance likely reflects historical introduction and successful local expansion, certain Mexican sub-lineages, especially X3, carry a higher burden of resistance and warrant continued genomic surveillance. Surprisingly, 98% of our samples contain mutations classified as “not associated with resistance” in *gyrA* E21Q and 81% also carry S95T and G668D mutations, perhaps reflecting the frequent use of fluoroquinolones in the country.

The most frequent drug resistance-conferring mutations identified in our dataset have already been reported elsewhere (World Health Organization, 2023; The CRyPTIC Consortium, 2022), and include *katG*_S315T, *rpoB*_S540L, and *embB*_D354A. The similarity between previous reports in Mexico and WHO global data reflects common adaptative mechanisms against anti-TB drugs while maintaining fitness, suggesting that existing genotyping tests that include these mutations remain useful for drug-resistant prediction and disease management.

However, we did identify novel potential resistance-conferring mutations: 89 variants in 30 genes and promoters; they include polymorphisms in genes previously associated with resistance, including *katG* for isoniazid, *embB* for ethambutol, *gyrA* and *gyrB* for fluoroquinolones, and *rrs* for aminoglycosides, as well as others in genes so far not associated with resistance.

Among these, we found 18 variants on *ahpC*. The *ahpC* gene encodes alkyl hydroperoxide reductase C, which is part of the bacterial antioxidant defense system. The AhpC protein helps protect against oxidative damage produced by isoniazid, thus variants in *ahpC* can serve as compensatory changes that help bacteria survive the oxidative stress generated by isoniazid activity. Although none of the specific variants have been associated with resistance, changes in the expression or activity of AphC may increase resistance to isoniazid, which is why WHO considers them as secondary mutations and labeled as “candidate resistance genes” (World Health Organization, 2023).

Another interesting example is the variant found in the *dprE2* gene. *dprE2* is part of an operon that encodes *dprE1-E2*, which are essential for the synthesis of the arabinogalactan and lipoarabinomannan components of the bacterial cell wall. Recently (Abrahams et al., 2023), DprE2 was identified as the target of activated Pretomanid and Delamanid. Although not yet validated, it is identified as a potential antimycobacterial target of these drugs and thus susceptible to resistance. All these variants should be validated with phenotypic tests, and may provide new insight into resistance variants in the region.

Additionally, we found that 40.9% (9 out of 22) of *rpoB*_S450L mutations had compensatory mutations in *rpoC*, which has been reported to improve the fitness of an *rpoB* rifampicin-resistant strains and associated with resistance outbreaks (Comas et al., 2012; Conkle-Gutierrez et al., 2023). Consistent with these findings, four of these strains were associated with poor clinical outcomes, and two samples had an increased resistance profile. However our numbers are too small to do proper statistical analysis.

We found a high level of concordance between genotypic and phenotypic analysis in strains containing the most common resistant variants, and a lower concordance when all the samples are included. The discrepancy between genotypic and phenotypic analysis is not particular to our study (Mejía-Ponce et al., 2023; Hasan et al., 2024). Although phenotypic tests are still considered the “golden standard,” it is also subject to interpretation and execution. Critical concentration values have changed through the years; specifically, tests for some of our samples were conducted before 2021, with altered critical concentration values for rifampicin, isoniazid, and fluoroquinolones, or no microbiological test for certain drugs. Furthermore, more analysis is still needed to determine all the specific genes involved in resistance to newer drugs such as bedaquiline, linezolid, delamanid, etc., and whether they vary by region or previous treatments.

Additionally, we identified five strains with heteroresistance: three strains to fluoroquinolones, one to rifampicin, and one to a combination of rifampicin, isoniazid, and pyrazinamide. Although we do not have the therapeutic outcome for all these patients, the majority of them had poor clinical outcomes. Heteroresistance could reveal the inherent complexity of resistance evolution and possible epistatic effects and must be further analyzed.

On the other hand, efflux pumps are emerging as critical players in antibiotic resistance not only in tuberculosis but in all infectious diseases. Efflux pumps represent a critical intersection between essential cellular maintenance systems and barriers to antimicrobial therapy. The presence of multiple efflux pumps in diverse bacteria, including Gram positive and Gram negative genera and fungi, is associated with increased antibiotic resistance (Adams et al., 2021; Han et al., 2024; Leconte et al., 2024; Li et al., 2024). The vast majority of the variants on efflux pumps found in our sample population are on Mycobacterial membrane protein Large (MmpL) genes; 22 on *mmpL8*, 12 on *mmpL3*, followed by ABC transporter Rv1458c with 18 variants. MmpL proteins are essential for cell wall biosynthesis and lipid transport, which also transport antibiotic compounds out of the cell. MmpL8 is required for biosynthesis and transport of SL-1 sulfated glycolipid that is involved in host-pathogen interactions during early infections. MmpL3 is key for trehalose monomycolate transport, an indispensable component of the mycobacterial cell wall. MmpL3 is the target of an ethambutol analog, SQ109, which is now in phase 2 clinical trials (Chaitra et al., 2023).

Both by modifying the impermeability of the cell wall and exporting antibiotic compounds, efflux pumps can work synergistically with other resistance mechanisms to increase antimicrobial resistance. Furthermore, several studies have demonstrated that antibiotic treatment upregulates these integral membrane proteins, thereby maintaining sublethal intracellular antibiotic concentrations and selecting for antibiotic-resistant mutants (Hao et al., 2011; Laws et al., 2022; Remm et al., 2022). Thus, the administration of inhibitors may play an important role in enhancing the efficacy of antibiotics in the treatment of bacterial and fungal infections (El Meouche and Dunlop, 2018; Grimsey et al., 2020; de Melo Guedes et al., 2024; Han et al., 2024; Leconte et al., 2024; Li et al., 2024; Ye et al., 2024). In particular, the use of verapamil, chlorpromazine, reserpine, and other efflux pump inhibitors, as adjunct therapy in the treatment of TB and other infectious diseases is a promising strategy to combat antibiotic resistance (Rodrigues et al., 2020; Tran et al., 2020; Remm et al., 2022; Rao and Bhosale, 2024).

In this study, we found that 73% of the analyzed strains had efflux pump variants, the majority of which belonged to the ABC and RND superfamilies, previously associated with antibiotic resistance (Anthony Malinga and Stoltz, 2016; Laws et al., 2022; The CRyPTIC Consortium, 2022; Hasan et al., 2024). It is important to follow these patients through their treatment, as variations in efflux pumps may be the origin of resistance before fixation of mutations in drug targets (Laws et al., 2022). Future research on antibiotic resistance must include efflux pumps to gain a comprehensive understanding of resistance.

Our study has several limitations, including the limited number of samples and the fact that DNA extraction of all our samples was performed on the first subculture after Mtb isolation from the patient, which may have altered the resistant populations. Additionally, not all strains have complete first and second-line pDST. Future studies must include pDST for the new variants found; furthermore, we think that following patients with efflux pump mutations throughout their treatment can shed light into the connection between these proteins and the development of resistance.

In this study, we identified several of the most prevalent mutations conferring antibiotic resistance in patients with TB, as well as new variants that require validation in drug susceptibility tests to assess their relevance to resistance. This study substantially increases the national variant registry and, together with phenotypical drug sensitivity testing, provides valuable insight into the epidemiological landscape of the country, specifically for Pre-XDR and XDR strains. Additionally, we identified variants in efflux pumps, which may be part of the resistance mechanism that needs further investigation.

Furthermore, our findings confirm the need for timely and comprehensive drug susceptibility testing combined with WGS analysis before initiating treatment, which in Mexico, has unfortunately relied primarily on empirical approaches until now. As sequencing becomes more accessible, WGS should become standard practice in both diagnostic and follow-up protocols. This technology enables precise identification of all drug resistance mutations, which is particularly critical for Pre-XDR and XDR tuberculosis strains where treatment options are already limited. The comprehensive resistance profiling will allow clinicians to design personalized treatment regimens based on each patient’s specific resistance profile, moving away from empirical therapy. This precision is especially important for extensively drug-resistant cases, where selecting inappropriate drugs can significantly worsen patient outcomes. Additionally, WGS helps preserve the effectiveness of remaining active drugs—such as bedaquiline, delamanid, pretomanid and linezolid by ensuring they are used appropriately and in optimal combinations to prevent further resistance development.

## Data Availability

The datasets presented in this study can be found in online repositories. The names of the repository/repositories and accession number(s) can be found in the article/Supplementary material.

## Footnotes

^1^www.mykrobe.com, Predictor version v0.10.0
