# Supplementary material for: Mycobacterium tuberculosis genomic surveillance in Mexico. Characterization of variants in drug resistance and efflux pump genes
Source: Front Microbiol. 2025 Oct 15;16:1666838. doi: 10.3389/fmicb.2025.1666838 (PMC12568502; doi:10.3389/fmicb.2025.1666838)
Supplement: Supplementary file 1 [file Table_1.docx]

**Sup.Table 1 . Mutations not associated with resistance.**

Variants found in genes previously associated with resistance, reported as not associated with resistance.

| **Drug** | **Gen** | **Variants** | **The number of strains with this variant** |
| --- | --- | --- | --- |
| Isoniazid | *katG* | R463L | 2 |
|  | *ndh* | V18A | 8 |
|  | *mshA* | N111S | 11 |
|  | *dnaA* | P124L | 7 |
|  |  | H156R | 1 |
| Rifampicin | *rpoC* | G594E | 26 |
| Ethambutol | *embB* | V668I | 2 |
|  |  | N13S | 2 |
|  |  | E378A | 2 |
|  | *embC* | V981L | 24 |
|  |  | T270I | 1 |
|  | *ubiA* | E149D | 1 |
| Levofloxacin | *gyrA* | E21Q | 48 |
|  |  | S95T | 40 |
|  |  | G668D | 40 |
|  | *gyrB* | A403S | 2 |
| Moxifloxacin | *gyrA* | E21Q | 48 |
|  |  | S95T | 40 |
|  |  | G668D | 40 |
| Amikacin | *rrs* | 517C>T | 1 |
| Streptomycin | *gid* | L16R | 1 |
