# Supplementary material for: Mycobacterium tuberculosis genomic surveillance in Mexico. Characterization of variants in drug resistance and efflux pump genes
Source: Front Microbiol. 2025 Oct 15;16:1666838. doi: 10.3389/fmicb.2025.1666838 (PMC12568502; doi:10.3389/fmicb.2025.1666838)
Supplement: Supplementary file 2 [file Table_2.docx]

**Sup. Table 2 . Mutations not reported on databases and mutations with uncertain significance.**

Variants not reported associated with resistance and reported as uncertain association, found in genes previously associated with resistance.

| **Drugs** | **Gen** | **Variants** | **The number of strains with this variant** |
| --- | --- | --- | --- |
| Isoniazid | *katG* | M257I | 2 |
|  |  | G421A | 2 |
|  |  | A379V | 2 |
|  |  | T86P | 1 |
|  |  | W438G | 1 |
|  |  | V320L | 1 |
|  |  | G124fs | 1 |
|  |  | I524N | 1 |
|  |  | W204* | 1 |
|  | *ahpC* | D73H | 12 |
|  |  | 81C>T | 2 |
|  |  | 52C>A | 2 |
|  |  | 51G>A | 1 |
|  |  | 47_-46insT | 1 |
|  | *inhA* | I21T | 1 |
|  | *dnaA* | Q71E | 1 |
|  |  | L250I | 1 |
|  | *ndh* | R284W | 1 |
|  |  | I212fs | 1 |
|  |  | I212L | 1 |
|  | *mshA* | R253L | 1 |
|  | *furA* | H118fs | 1 |
| Rifampicin | *rpoB* | A753V | 2 |
|  |  | L957P | 1 |
|  |  | A1152V | 1 |
|  | *rpoC* | V483G | 6 |
|  |  | I491V | 2 |
|  |  | I851V | 1 |
|  |  | D747G | 1 |
|  |  | V483A | 1 |
|  |  | P1040A | 1 |
| Ethambutol | *embB* | D1024N | 2 |
|  |  | D1041A | 1 |
|  | *embA* | A409T | 2 |
|  |  | G230S | 1 |
|  |  | V911A | 1 |
|  |  | P969S | 1 |
|  | *embC* | T669A | 1 |
|  | *ubiA* | G165S | 1 |
| Pyrazinamide | *pncA* | V157G | 6 |
|  |  | T135A | 1 |
|  |  | D136fs | 1 |
|  | *rpsA* | A440T | 3 |
|  |  | I55T | 1 |
|  |  | E154K | 1 |
| Streptomycin | *gid* | A138V | 6 |
|  |  | G71fs | 3 |
|  |  | S149R | 2 |
|  |  | G164D | 2 |
|  |  | S136P | 2 |
|  |  | L90R | 1 |
|  |  | G76D | 1 |
|  |  | L16P | 1 |
|  |  | K163N | 1 |
|  |  | P75S | 1 |
|  |  | L79S | 1 |
| Amikacin | *eis* | T70A | 2 |
|  | *rrs* | 1473084T>C | 2 |
|  |  | 1472868_1472869insCGG | 1 |
|  |  | 1472872_1472873delGC | 1 |
|  |  | 1472880_1472881insA | 1 |
|  |  | 1472953_1472954insA | 1 |
| Levofloxacin | *gyrA* | D834N | 4 |
|  |  | D639A | 1 |
|  |  | E214G | 2 |
|  |  | P124A | 1 |
|  |  | A288D | 1 |
|  | *gyrB* | A238P | 1 |
| Moxifloxacin | *gyrA* | D834N | 4 |
|  |  | D639A | 1 |
|  |  | E214G | 2 |
|  |  | P124A | 1 |
|  |  | A288D | 1 |
|  | *gyrB* | A403S | 2 |
|  |  | A238P | 1 |
| Linezolid | *rrl* | 1474001C>T | 8 |
| Cycloserine | *aldC* | K186N | 9 |
|  |  | T21A | 7 |
|  |  | L244P | 1 |
| Etionamide | *ethA* | Q291* | 6 |
|  | *ethR* | P163P | 1 |
|  |  | *17G>C | 1 |
| Para-aminosalicylate sodium (PAS) | *thyA* | T202A | 2 |
|  |  | A168D | 2 |
| Delamanid | *dprE2* | D45N | 7 |
|  | *fbiA* | I208V | 1 |
|  | *fbiB* | G19E | 1 |
|  | *ddn* | 18G>A | 1 |
| Clofazimine | *mmpR5* | I67fs | 1 |
| **Mutations with uncertain significance** | | | |
| Isoniazid | *katG* | A480del | 1 |
| Ethambutol | *embA* | 12C>T | 4 |
